# Trans-Perineal Template-Guided Mapping Biopsy vs. Freehand Trans-Perineal Biopsy in Chinese Patients With PSA < 20 ng/ml: Similar Cancer Detection Rate but Different Lesion Detection Rate

**DOI:** 10.3389/fonc.2019.00758

**Published:** 2019-08-09

**Authors:** Bi-Ming He, Rui Chen, Zhen-Kai Shi, Guang-An Xiao, Hu-Sheng Li, Heng-Zhi Lin, Jin Ji, Hong-Xiang Peng, Yan Wang, Ying-Hao Sun, Hai-Feng Wang

**Affiliations:** Department of Urology, Shanghai Changhai Hospital, Second Military Medical University, Shanghai, China

**Keywords:** biopsy, trans-perineal, template, freehand, cancer detection, lesion detection

## Abstract

The present study aimed to investigate the diagnostic efficacy and the regional location of prostate cancer (PCa) as well as the accuracy of assessment between trans-perineal template-guided mapping biopsy (TTMB) and freehand trans-perineal biopsy (FTPB) for men with PSA < 20 ng/ml. Thus, we evaluated 623 consecutive patients with PSA < 20 ng/ml who had prostate biopsies in our institute between July 2017 and September 2018. Patients were divided into two groups based on different biopsy methods: 217 (34.83%) patients with TTMB and 406 (65.17%) with FTPB. Thirty six patients with TTMB and 80 with FTPB had continued undergone radical prostatectomy after a cancer diagnosis. Then the Gleason score of the biopsy and the post-radical prostatectomy specimens in each patient were compared. Overall, the PCa detection rate was 34.35%. There was no significant difference in PCa detection rate between TTMB and FTPB (35.48 vs. 33.74%, respectively; *p* = 0.663). Besides, the detection rate of significant PCa (Gleason score ≥ 7) in TTMB was 29.03% while FTPB was 23.89% (*p* = 0.162). The detection rate at the apex of the prostate was higher than the detection rate at the base of the prostate (9.80 vs. 5.79%; *p* < 0.01) when performing the TTMB. The FTPB would miss 10% of the positive diagnosis and almost half of the lesions. The upgraded of Gleason score from biopsy to post-radical prostatectomy was 16.67% with the TTMB and 36.25% with the FTPB (*p* = 0.034). The TTMB had a similar cancer detection rate, but a higher lesion detection rate and more accuracy in assess the actual Gleason score when comparing to FTPB for men with PSA < 20 ng/ml. By performing a 20-core TTMB, the cancer detection rate at the apex of the prostate was higher than the base.

## Introduction

Prostate cancer (PCa) is the second most prevalent carcinoma and the fifth leading cause of death in males worldwide (1). Cancer detection is crucial for the management of the malignancy to make the medical decision and reduce mortality. Although serum PSA level, digital rectal examination (DRE), transrectal ultrasound (TRUS), and magnetic resonance imaging (MRI) help a lot for PCa detection, the prostate biopsy is still the “gold standard” for confirming the histological diagnosis for now.

Since 6-core biopsy was first introduced by Hodge et al. (2), the technique of biopsy had noticeably improved. Currently, trans-rectal biopsy becomes the most popular means in PCa diagnosis; however, this method has drawbacks for its limitation to evaluate the entire prostate gland (3), and its moderately high infectious complications which including urinary tract infection, prostatitis, and sepsis (4). Compared with trans-rectal biopsy, the trans-perineal biopsy (TPB) is considered to have higher cancer detection at the anterior and apex of the prostate (5, 6) but a lower rate of infection (7). Besides, it also provided more accurate prediction in determining final Gleason score and clinical risk category (8).

TPB was originated as a freehand TPB (FTPB) procedure, using just a biopsy gun and a TRUS probe. It may be convenient and time-saving for an experienced urologist; however, it is a technique which is difficult to access for guiding the needle to get the target region of the prostate and controlling puncture direction. Trans-perineal template-guided mapping biopsy (TTMB), with a brachytherapy stepping unit and grid, on the contrary, makes it easy to drive the needle for sampling the prostate. However, the equipment is expensive and the biopsy procedure takes more time. To now, there is limited literature regarding the comparison of cancer detection between TTMB and FTMB.

We present a retrospective study of 623 consecutive patients with PSA < 20 ng/ml who had a prostate biopsy in our institute. A 12-core FTMB was adopted in our institute on July 2017 while the biopsy protocol changed to a 20-core TTMB in March 2018 for screening the men who are potentially suitable for focal therapy. In the present study, patients were divided into two groups: those with TTMB and those with FTPB. We evaluated the detection rate and the regional location of cancer in different biopsy methods as well as the accuracy of Gleason score between biopsy and radical prostatectomy specimen. The objective of this study is to find out if there be a difference between TTMB and FTPB biopsy in cancer detection and lesion detection.

## Patients and Methods

### Patient Population

This was a retrospective study conducted by us in Shanghai Changhai Hospital, Second Military Medical University. Patients whose serum PSA level was <20 ng/ml, and who had undergone the prostate biopsy in our institute between July 2017 and September 2018 were included. Those who with a history of prostate cancer treatment (radiotherapy, focal therapy, or endocrine) before biopsy were excluded. Patients were selected from 2 main clinical cohorts, including those with TTMB (from March 2018 to September 2018) and those with FTPB (from July 2017 to March 2018). The study was conducted by the principles of the Declaration of Helsinki, and the study protocol was approved by the research ethics committee of Changhai Hospital, Shanghai, China. Because of the retrospective nature of the study, patient consent for inclusion was waived.

### Procedures

**Figure 1:**
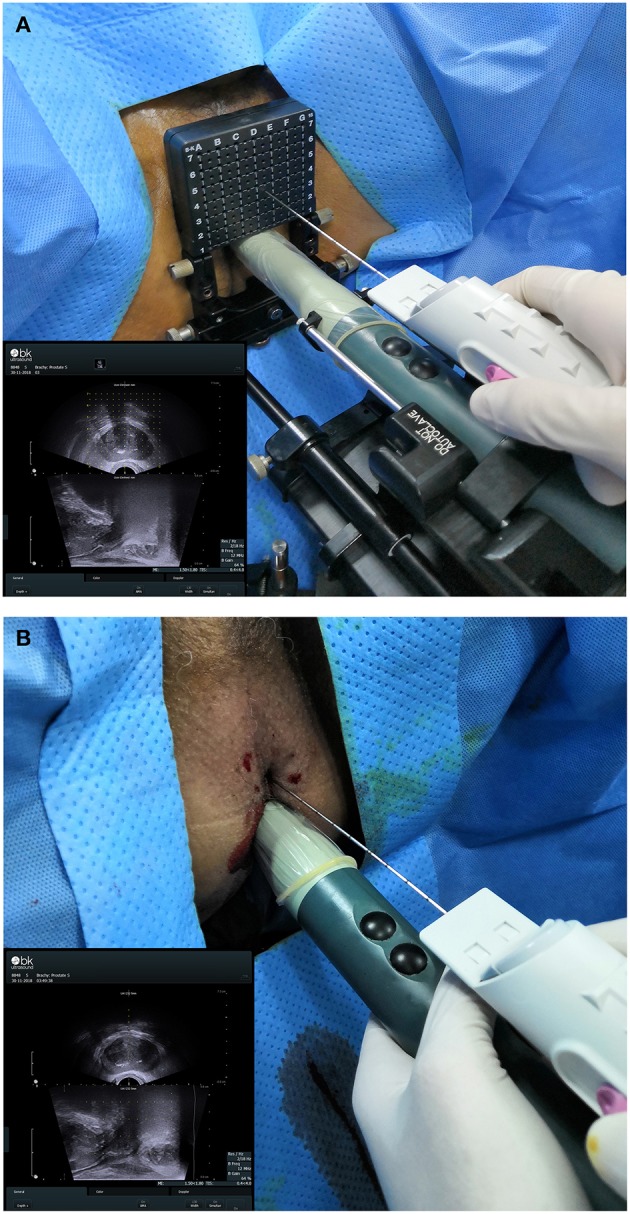
**(A)** Trans-perineal template-guided mapping biopsy. **(B)** Freehand trans-perineal biopsy.

20-core TTMB and 12-core FTPB were performed via perineal with local anesthesia. An UltraView 800 ultrasound device (BK Ultrasound, USA) equipped with a bi-planar TRUS probe (8848, BK Ultrasound) and a biopsy gun (Magnum MG15-22; Bard, USA) equipped with a biopsy needle (18 G, 130; Bard) was used (Figure 1). The standard 5 mm template brachytherapy grid was preparing for the TTMB. The biopsy region protocol of TTMB was referenced to Singh et al. (9) while FTPB was to Guo et al. (10). All biopsied were performed by three experienced surgeons (one experience more than 10 years and two experience more than 5 years). After a biopsy, part of patients (*n* = 116) with prostate cancer had received radical prostatectomy in our institute. The biopsy and the specimens in each patient would be compared. Both biopsy and post-radical prostatectomy specimens were assessed by the same pathology group. Two pathologists in the group had more than 10 years of experience and blind to previous biopsy results. The significant cancer was defined as Gleason score ≥ 7.

### Statistical Analyses

Clinical characteristic data were described using median and interquartile range (IQR), biopsy data were described using number (n) and percentage (%). The detection rates were compared using the chi-squared test. All reported *P*-values were two-sided, and the statistical significance was considered at *P* < 0.05. The statistical analysis was performed using SPSS19.0 (Chicago, IL, USA) and the figure was performed using Cinema 4D R19 and Adobe Illustrator CC 2017.

## Results

### Patient Characteristics

A total of 623 patients were involved in this study, of which 217 had undergone TTMB, while 406 had the FTPB. The clinical characteristics of patients with TTMB and those with FTPB shown in Table 1. The median[interquartile range(IQR)] age, BMI, serum PSA level, prostate volume, and PSA density value were 66(10.5) years, 23.94(3.74), 8.60(5.79) ng/ml, 39.88(30.65) ml, and 0.23(0.22) ng/ml/ml in men with TTMB and 66(9) years, 24.22(3.76), 8.75(5.9) ng/ml, 39.79(28.39) ml, and 0.26(0.24) ng/ml/ml in men with FTPB, respectively.

**Table 1:** Clinical characteristics of the patients included in the study.

**Variable**	**Overall cohort**	**Template-guided biopsy**	**Freehand biopsy**
Patients NO.	623	217	406
Age, yr (median, IQR)	66 (9)	66 (10.5)	66 (9)
BMI (median, IQR)	24.22 (3.76)	23.94 (3.74)	24.22 (3.76)
PSA, ng/ml (median, IQR)	8.72 (5.86)	8.60 (5.79)	8.75 (5.9)
Prostate volume, ml (median, IQR)	39.79 (30.16)	39.88 (30.65)	39.79 (28.39)
PSAD, ng/mlper gram (median, IQR)	0.25 (0.22)	0.23 (0.22)	0.26 (024)

*BMI, Body Mass Index; PSA, prostate specific antigen; PSAD, PSA density*.

### Biopsy Outcome

In all, 214(34.55%) of the 623 patients were diagnosed with prostate cancer, including 77(35.48%) of them with TTMB and 137(33.74) with FTPB, respectively (Table 2a). Within the patients with prostate cancer, 74.77% (160/214) were clinically significant; 29.44% (63/214) in the cohort of TTMB and 45.33% (97/214) in the cohort of FTPB (Table 2b). In the overall patient pool, the number of cancer cores was 927(10.06%), for 373(8.59%) with TTMB and 554(11.37%) with FTPB (Table 2c). In addition, TTMB detected 14 patients who had Gleason score 6, 34 Gleason score 3+4, 8 Gleason score 4+3, 15 Gleason score 8, 5 Gleason score 9, and 1 Gleason score 10 while FTPB detected 40 Gleason score 6, 35 Gleason score 3+4, 11 Gleason score 4+3, 24 Gleason score 8, 20 Gleason score 9, and 7 Gleason score 10, respectively (Tables 2d-2i). The negative biopsy was 140(64.52%) for TTMB and 269(66.26%) for FTPB (Table 2j).

**Table 2:** Comparison of biopsy result by template-guided mapping biopsies and freehand systematic biopsies.

**Variable**	**Overall cohort**	**Template-guided biopsy**	**Freehand biopsy**
a.Overall PCa, *n* (%)	214 (34.35)	77 (35.48)	137 (33.74)
b.Significant PCa, *n* (%)	160 (25.68)	63 (29.03)	97 (23.89)
c.Number of cancer cores, *n* (%)	927 (10.06)	373 (8.59)	554 (11.37)
d.Gleason Score 6, *n* (%)	54 (8.67)	14 (6.45)	40 (9.85)
e.Gleason Score 3 + 4, *n* (%)	69 (11.08)	34 (15.67)	35 (8.62)
f. Gleason Score 4 + 3, *n* (%)	19 (3.05)	8 (3.69)	11 (2.71)
g.Gleason Score 8, *n* (%)	39 (6.26)	15 (6.91)	24 (5.91)
h.Gleason Score 9, *n* (%)	25 (4.01)	5 (2.30)	20 (4.93)
i.Gleason Score 10, *n* (%)	8 (1.28)	1(0.46)	7 (1.72)
j.NBx, *n* (%)	409 (65.65)	140 (64.52)	269 (66.26)

*Pca, Prostate Cancer; Nbx, negative biopsy*.

### Detection Rate of Prostate Cancer

The overall detection rate of PCa between the cohort of TTMB and the cohort of FTPB did not differ significantly (*p* = 0.663; Table 3a). Although the clinically significant PCa detection rate with TTMB was higher than the FTPB (29.03 vs. 23.89%), the *p*-value still showed no significant difference (Table 3b). There was also no difference in different ranges of age (38.46 vs. 15.66% in age <60 years; *p* = 0.124, 33.62 vs. 34.89% in age 60–70 years; *p* = 0.813 and 43.08 vs. 47.19% in age > 70 years; *p* = 0.613 respectively, Table 3c). In addition, there was no difference between these two cohorts when serum PSA level (31.85 vs. 29.84% in PSA < 10 ng/ml; *p* = 0.683 and 41.46 vs. 39.87% in PSA 10–20 ng/ml; *p* = 0.812, Table 3d) or prostate volume (62.96 vs. 61.25% in prostate volume < 40 ml; *p* = 0.841 and 25.93 vs. 26.58% in prostate volume ≥ 40; *p* = 0.537, Table 3e) were examined.

**Table 3:** Comparison of detective rate of prostate cancer of template-guide mapping biopsy and freehand systematic biopsy.

**Positive Rate**	**Overall cohort**	**Template-guided biopsy**	**Freehand biopsy**	***P*-value**
Patients included, a-e	623	217	406	
a.overall PCa, %	34.35	35.48	33.74	0.663
b.Significant PCa, %	25.68	29.03	23.89	0.162
**c.Age, %**				
<60	15.66	38.46	15.66	0.124
60–70	34.47	33.62	34.89	0.813
>70	45.75	43.08	47.19	0.613
**d.PSA, %**				
<10	30.55	31.85	29.84	0.683
10-20	40.42	41.46	39.87	0.812
**e.Prostate volume, %**				
<40	61.94	62.96	61.25	0.841
≥40	26.32	25.93	26.58	0.537

*P < 0.05 is statistically significant*.

### Biopsy Region and Lesions Detection

**Figure 2:**
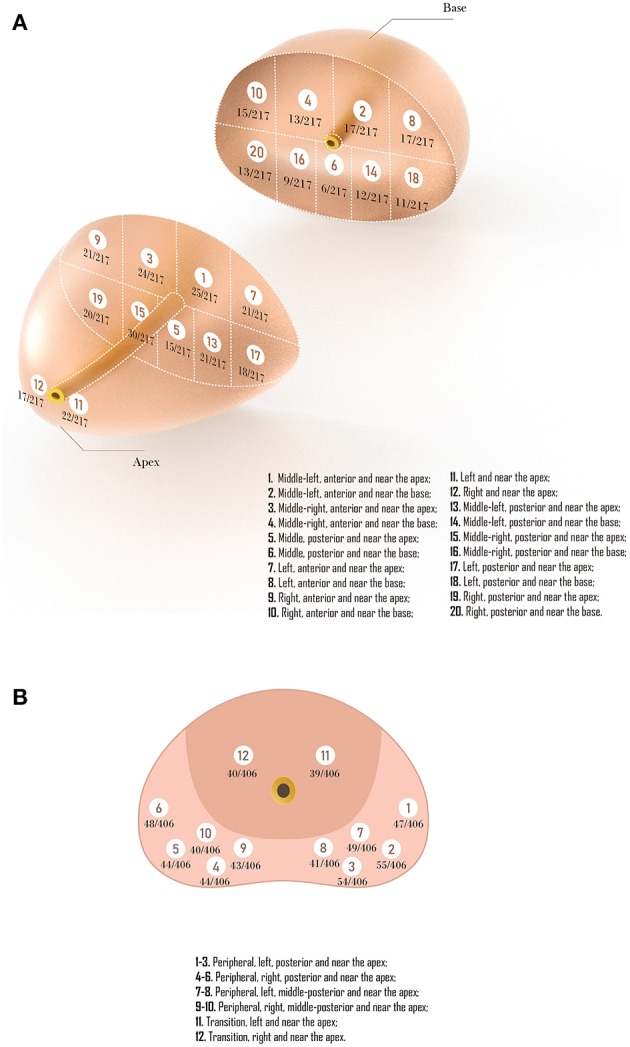
**(A)** Representative trans-perineal template-guided mapping biopsy regions. **(B)** Freehand trans-perineal biopsy regions.

The results of TTMB regions are shown in Figure 2A. The number of biopsy cores for all 217 patients added up to 4,340, of which 2,387 were in the region from the apex to the mid-gland, and 1,953 were in the region from mid-gland to the base. Within these cores, 347 were detected cancer. Nearly two-thirds of PCa (208/347, 59.94%) were found in the peripheral zone (region 7–10 and region 11–20), and 11.24% (39/347) were in the apex. Also, there were 44.09% (153/347) in the anterior sector lesions (region 1–4, 7–10) while 44.67% (155/347) in the posterior sector (region 5–6, 13–20). The detection rate of PCa in the sector from the apex to the mid-gland (9.80%, 234/2387) was higher than the sector from mid-gland to the base (5.79%, 113/1953). Moreover, the central zone under urethra (region 5–6) had the lowest detection rate (4.84%) compared with other lesions. For FTPB, a total of 544 positive cores were detected, of which 465(85.47%) in the peripheral zone (region 1–10), and 79 (14.52%) were in the transition zone (region 11–12; Figure 2B).

**Figure 3:**
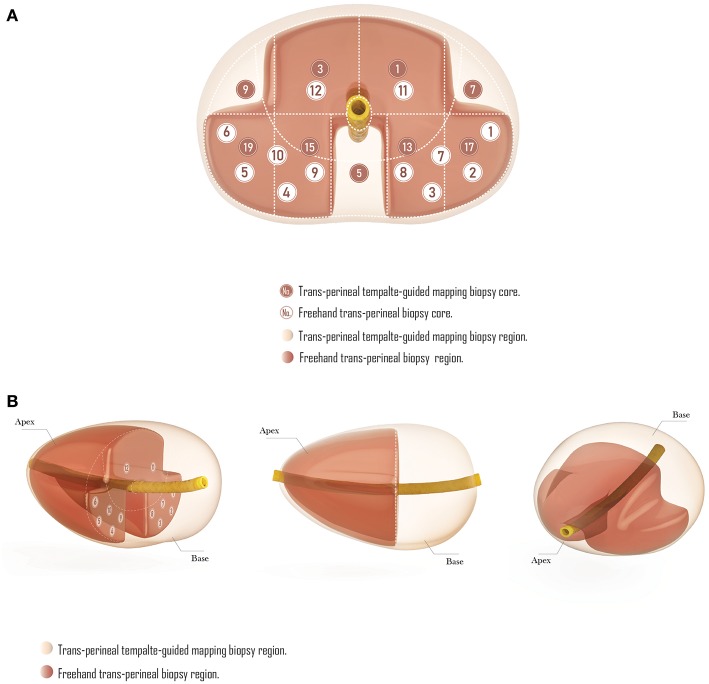
The comparison of trans-perineal template-guided mapping biopsy and freehand trans-perineal biopsy. **(A)** Coronal plane. **(B)** Perspective view.

Compared with TTMB, the FTPB lacks biopsy cores in the central zone under urethra (region 5 in template-guided biopsy), the left and right anterior sector (region 7, 9 in TTMB), and the sector from mid-gland to the base (region 2, 4, 6, 8, 10, 14, 16, 18, 20 in TTMB; Figure 3). Nine patients (11.7%) were found a single lesion in these regions with TTMB, which indicated that the outcome would be negative if they have undergone the 12-core freehand biopsy. Also, 170 lesions were detected from these regions, which accounted for 49% of the whole (*n* = 347).

### Comparison of the Biopsy and Radical Prostatectomy Gleason Score

The radical prostatectomy Gleason score of patients with TTMB and those with FTPB are shown in Table 4. The final pathologic Gleason score was upgraded in 6 (16.67%) cases in patients with TTMB and 29 (36.25%) cases among men with FTPB (*p* = 0.034). A total of 20 (55.56%) men with TTMB and 30 (37.50%) of FTPB patients had the same Gleason score at biopsy and radical prostatectomy (*p* = 0.069). Nevertheless, 10 (27.78%) patients with the TTMB and 20 (25%) patients with the TTMB showed downgrading of the Gleason score from biopsy to radical prostatectomy (*p* = 0.820).

**Table 4:** Comparison of biopsy and radical prostatectomy Gleason scores in patients with prostate cancer.

**Template-guided biopsy Gleason Score**	**RP Gleason Score**						**Total number**
	**6**	**3 + 4**	**4 + 3**	**8**	**9**	**10**	
6	1	3	0	0	0	0	4
3 + 4	1	13	0	1	0	0	15
4 + 3	1	2	3	1	0	0	7
8	0	2	3	2	1	0	8
9	0	0	1	0	1	0	2
10	0	0	0	0	0	0	0
Total number	3	20	7	4	2	0	36
**Freehand biopsy Gleason Score**	**RP Gleason Score**						**Total number**
	6	3 + 4	4 + 3	8	9	10	
6	5	16	5	0	0	0	26
3 + 4	1	15	5	0	0	0	21
4 + 3	0	3	4	1	1	0	9
8	1	3	9	1	1	0	15
9	0	0	2	1	5	0	8
10	0	0	0	0	1	0	1
Total number	7	37	25	3	8	0	80

*RP, radical prostatectomy*.

## Discussion

The cancer detection rate in the TTMP cohort is 35.48%, while FTPB is 33.74%, and the detection rate for significant cancer is 29.03 and 23.89%, respectively. The detection rates obtained in the PROMIS study, showed 71% for cancer and 57% for significant cancer when performing the TTMP (11). Another technique of FTPB reported by Ristau et al. reporting on 1,000 men, demonstrated that the cancer detection rate is 60.7% and significant cancer is 40.3% (12). The detection rate of TTMP and FTPB in our study is lower than the prior's study. We consider that the differences are caused by the incidence of prostate cancer in the different country/region. The data from GLOBOCAN 2018 demonstrated the incidence of PCa is 13.9% for Eastern Asia (our study), 75.8% for Western Europe (PROMIS study), and 73.7% for Northern America (Ristau et al. study) (1). Our study showed that TTMB and FTPB had similar performance in cancer or significant cancer detection. For reducing bias, we also categorized patients based on different baseline data such as yeas, PSA or prostate volume. However, TTMB and FTPB still remained similar performance in cancer detection for classified men.

Although the TTMB was performed with more biopsy cores when comparing to the FTPB (20 vs. 12) in the present study, it failed to show an improvement in detection rate. Previous studies have shown evidence to support this opinion. Scattoni et al. confirmed no significant change in detection between 18-core and 12-core biopsy as the rate of 39.9 and 38.4%, respectively (13). Similarly, Abd et al. demonstrated no difference in detection when comparing 12-core (49.2%) to 8-core (51.2%) biopsy (14). We considered the cause of these outcomes is related to the biopsy region. When biopsy with fewer cores concentrates more on regions with higher detection rates, cancer detections are thus increased and equivalent to the biopsy with more cores.

In our study, although the biopsy region of TTMB was almost evenly distributed in part from apex to mid-gland and the part from mid-gland to the basal sector, the detection rate of positive cores near the apex was nearly 1.7 times more than the basal sector (234/2387 vs. 113/1953; *p* < 0.01). As a comparison, all biopsy regions of FTPB were located close to the apex of the prostate. It seems that the biopsy region distribution of the two methods contributes a lot for their detection rate. Compared with TTMB, the FTPB had less count but more effective cores.

The different distribution of biopsy regions helped FTPB to have similar cancer detection rate as TTMB, with fewer but more efficient cores, however, for this reason, it also led to the less detected lesions with FTPB. Besides, in the detection of lesions in different regions of the prostate, TTMB easily missed the lesions which under the urethra, the left and right anterior, and the base, even though the incidence of lesions in these regions may not as high as the peripheral zone, the transition zone, and the apex. Though only nine patients (11.7%) had been detected a single lesion in these regions, the number of overall detected lesions in these regions (*n* = 170) accounted for 49% within all 347 lesions. That is to say, compared with the TTMB, the FTPB will only miss 10% of the positive diagnosis, but it will miss almost half of the lesions.

In addition to detecting tumors, the evaluation of the Pathological grade is also a critical goal of prostate biopsy. Based on the malignancy degree, the Gleason score system was widely used to grade PCa. However, there is always a difference in Gleason score between biopsy and radical prostatectomy, for the problems inherent with biopsy sampling. From the previous study, about 30–40% of Gleason score in men who undergo radical prostatectomy are confirmed to be miss patched after the pathological review of the biopsy (15). In our research, compared with the post-radical prostatectomy specimen, the compatibility in Gleason score of TTMB is 55.56% while the FTPB is 37.50% (*p* = 0.069). Besides, the final pathologic Gleason score was upgraded in 16.67% with the TTMB but 36.25% with FTPB (*P* = 0.034). It indicates that using the TTMB might assess the Gleason score of the prostate more accurately, as well as the FTPB, tend to underestimate Gleason score of the prostate. Although it may be a bias due to the different number of biopsy cores between TTMB and FTPB, the evidence from Moussa et al. (16) and Palisaar et al. (17) study showed no significant correlation between the number of biopsy cores and the final pathological upgraded.

With the emergence of treatment methods like focal therapy for PCa, especially localized PCa, the needed information from the biopsy is not only limited to the detection of tumors, but also the lesions of the tumors. The TTMB is superior to FTPB as a mean to localize individual PCa lesions, which is preparing for focal therapy, for its higher cores count and broader distribution. Also, with the standard 5 mm template grid providing more precise information, it helped a lot to mapped the exact location of each lesion in the prostate gland. Singh PB and his collaborators recommended the TTMB for patients selecting and tumor evaluation before performing a tissue-preserving focal therapy (9). Therefore, we consider the TTMB as a better choice for those with PSA < 20 ng/ml in our institute, for the potential alternative strategy as focal therapy instead of radical treatment, even though it might not be ascendant than the FTPB in cancer detection rate.

This study is not devoid of limitations. First, this was a retrospective study that suffers the drawbacks related to its nature. Second, this study lacks several factors for analyses, such as the percentage of the tumor and the length of the tumor in each core. Moreover, Although some of these cases were also performed an MRI-guided targeted biopsy (166 men with a MRI and 76 of these with a targeted biopsy combined with a TTMB or FTPB), we did not include the results of MRI and targeted biopsy in this study. We acknowledged that a targeted biopsy might have some influence on the TTMB or FTPB, which may lead to bias, even though the different biopsy procedure is independent of each other. Lastly, the data were collected from single-center, and the sample size of this study was limited.

In conclusion, TTMB and FTPB have a similar PCa detection rate in men with PSA < 20 ng/ml. The detection rate in each biopsy regions demonstrated that the number of positive cores near the apex of the prostate was more than the basal sector. The FTPB might miss 10% of patients with cancer and 49% of lesions for it lacks biopsy cores in some zone of the prostate, which regularly occurs in the TTMB. Besides, we found that TTMB might assess the Gleason score of the prostate more accurately, and less likely to be Gleason score upgrading from biopsy to post-radical prostatectomy specimens.

## Data Availability

The datasets generated for this study are available on request to the corresponding author.

## Ethics Statement

This retrospective study was approved by the research ethics committee of Changhai Hospital, Shanghai, China.

## Author Contributions

B-MH, Y-HS, and H-FW designed the study. B-MH, RC, Z-KS, G-AX, H-SL, H-ZL, JJ, H-XP, YW, Y-HS, and H-FW collected clinical data. B-MH, RC, Z-KS, G-AX, and H-SL analyzed and interpreted the data. B-MH, RC, Z-KS, and G-AX drafted and edited the manuscript. All authors read and approved the final version of the article.

### Conflict of Interest Statement

The authors declare that the research was conducted in the absence of any commercial or financial relationships that could be construed as a potential conflict of interest.

## References

[1] Bray F Ferlay J Soerjomataram I Siegel RL Torre LA Jemal A. Global cancer statistics 2018: GLOBOCAN estimates of incidence and mortality worldwide for 36 cancers in 185 countries. CA Cancer J Clin. (2018). 10.3322/caac.21492 30207593

[2] Hodge KK McNeal JE Terris MK Stamey TA. Random systematic versus directed ultrasound guided transrectal core biopsies of the prostate. J Urol. (1989) 142:71–4; discussion 4–5. 10.1016/S0022-5347(17)38664-0 2659827

[3] Seles M Gutschi T Mayrhofer K Fischereder K Ehrlich G Galle G. Sampling of the anterior apical region results in increased cancer detection and upgrading in transrectal repeat saturation biopsy of the prostate. BJU Int. (2016) 117:592–7. 10.1111/bju.13108 25726856

[4] Borghesi M Ahmed H Nam R Schaeffer E Schiavina R Taneja S. Complications after systematic, random, and image-guided prostate biopsy. Eur Urol. (2017) 71:353–65. 10.1016/j.eururo.2016.08.004 27543165

[5] Ayres BE Montgomery BS Barber NJ Pereira N Langley SE Denham P. The role of transperineal template prostate biopsies in restaging men with prostate cancer managed by active surveillance. BJU Int. (2012) 109:1170–6. 10.1111/j.1464-410X.2011.10480.x 21854535

[6] Mabjeesh NJ Lidawi G Chen J German L Matzkin H. High detection rate of significant prostate tumours in anterior zones using transperineal ultrasound-guided template saturation biopsy. BJU Int. (2012) 110:993–7. 10.1111/j.1464-410X.2012.10972.x 22394668

[7] Grummet JP Weerakoon M Huang S Lawrentschuk N Frydenberg M Moon DA. Sepsis and 'superbugs': should we favour the transperineal over the transrectal approach for prostate biopsy? BJU Int. (2014) 114:384–8. 10.1111/bju.12536 24612341

[8] Scott S Samaratunga H Chabert C Breckenridge M Gianduzzo T. Is transperineal prostate biopsy more accurate than transrectal biopsy in determining final Gleason score and clinical risk category? A comparative analysis. BJU Int. (2015) 116(Suppl 3):26–30. 10.1111/bju.13165 26260531

[9] Singh PB Anele C Dalton E Barbouti O Stevens D Gurung P. Prostate cancer tumour features on template prostate-mapping biopsies: implications for focal therapy. Eur Urol. (2014) 66:12–9. 10.1016/j.eururo.2013.09.045 24207133 PMC4062939

[10] Guo LH Wu R Xu HX Xu JM Wu J Wang S. Comparison between ultrasound guided transperineal and transrectal prostate biopsy: a prospective, randomized, and controlled trial. Sci Rep. (2015) 5:16089. 10.1038/srep16089 26526558 PMC4630643

[11] Ahmed HU El-Shater Bosaily A Brown LC Gabe R Kaplan R Parmar MK. Diagnostic accuracy of multi-parametric MRI and TRUS biopsy in prostate cancer (PROMIS): a paired validating confirmatory study. Lancet. (2017) 389:815–22. 10.1016/S0140-6736(16)32401-1 28110982

[12] Ristau BT Allaway M Cendo D Hart J Riley J Parousis V. Free-hand transperineal prostate biopsy provides acceptable cancer detection and minimizes risk of infection: evolving experience with a 10-sector template. Urol Oncol. (2018) 36:528.e15-528.e20. 10.1016/j.urolonc.2018.09.013 30446447

[13] Scattoni V Roscigno M Raber M Deho F Maga T Zanoni M. Initial extended transrectal prostate biopsy–are more prostate cancers detected with 18 cores than with 12 cores? J Urol. (2008) 179:1327–31; discussion 31. 10.1016/j.juro.2007.11.052 18289580

[14] Abd TT Goodman M Hall J Ritenour CW Petros JA Marshall FF. Comparison of 12-core versus 8-core prostate biopsy: multivariate analysis of large series of US veterans. Urology. (2011) 77:541–7. 10.1016/j.urology.2010.06.008 20817273

[15] Eggener SE Scardino PT Walsh PC Han M Partin AW Trock BJ. Predicting 15-year prostate cancer specific mortality after radical prostatectomy. J Urol. (2011) 185:869–75. 10.1016/j.juro.2010.10.057 21239008 PMC4058776

[16] Moussa AS Kattan MW Berglund R Yu C Fareed K Jones JS. A nomogram for predicting upgrading in patients with low- and intermediate-grade prostate cancer in the era of extended prostate sampling. BJU Int. (2010) 105:352–8. 10.1111/j.1464-410X.2009.08778.x 19681898

[17] Palisaar JR Noldus J Loppenberg B von Bodman C Sommerer F Eggert T. Comprehensive report on prostate cancer misclassification by 16 currently used low-risk and active surveillance criteria. BJU Int. (2012) 110:E172–81. 10.1111/j.1464-410X.2012.10935.x 22314081

